# Chronic Inflammatory Lameness Increases Cytokine Concentration in the Spinal Cord of Dairy Cows

**DOI:** 10.3389/fvets.2020.00125

**Published:** 2020-02-28

**Authors:** Daniel Herzberg, Pablo Strobel, Alfredo Ramirez-Reveco, Marianne Werner, Hedie Bustamante

**Affiliations:** ^1^Faculty of Veterinary Sciences, Graduate School, Universidad Austral de Chile, Valdivia, Chile; ^2^Faculty of Veterinary Sciences, Animal Science Institute, Universidad Austral de Chile, Valdivia, Chile; ^3^Faculty of Veterinary Sciences, Veterinary Clinical Sciences Institute, Universidad Austral de Chile, Valdivia, Chile

**Keywords:** dairy cows, lameness, chronic pain, cytokine, spinal cord

## Abstract

Lameness in dairy cows is an extremely painful multifactorial condition that affects the welfare of animals and economically impacts the dairy industry worldwide. The aim of this study was to determine the profile of cytokines in the spinal cord dorsal horn of dairy cows with painful chronic inflammatory lameness. Concentrations of 10 cytokines were measured in the spinal cord of seven adult dairy cows with chronic lameness and seven adult dairy cows with no lameness. In all cows lameness was evaluated using a mobility scoring system and registered accordingly. Immediately after euthanasia the spinal cord was removed and 20 cm of lumbar segments (L2–L5) were obtained. After dorsal horn removal and processing, cytokine quantification of tumor necrosis factor-alpha (TNF-α), interleukin-1alpha (IL-1α), interleukin 13 (IL-13), chemokine-10 (CXCL10/IP-10), chemokine-9 (CXCL9/MIG), interferon-alpha (IFN-α), interferon-gamma (IFN-γ), interleukin-21 (IL-21), interleukin-36ra (IL-36ra), and macrophage inflammatory protein-1 beta (MIP-1β) was performed using a multiplex array. Lame cows had higher concentrations of TNF-α, IL-1-α, IL-13, CXCL10, CXCL9, IFN-α, and IFN-γ in their dorsal horn compared to non-lame cows, while IL-21 concentration was decreased. No differences in IL-36ra and MIP-1β concentrations between lame and non-lame cows were observed. Painful chronic inflammation of the hoof in dairy cows leads to a marked increase in cytokine concentration in the dorsal horn of the spinal cord, which could represent a state of neuroinflammation of the Central Nervous System (CNS).

## Introduction

Intensive dairy farming may lead to negative health and welfare outcomes, including chronic painful lameness (1). Currently, chronic pain is recognized as a Central Nervous System (CNS) disease (2), and although no objective evidence has linked chronic pain with suffering in animals, we could not neglect the assumption that it may occur (3). Olsson et al. (4) indicate that animal suffering can be originated by exposure to different external or internal events that threaten biological functions. Chronic painful lameness significantly affects welfare (5) decreasing milk production and reproductive indexes, thus increasing early culling (6–8).

Cows with mild to severe lameness develop mechanical hyperalgesia of the dorsal aspect of the metatarsus (9), which is consistent with central sensitization, as secondary hyperalgesia is centrally and not peripherally mediated (10) and a positive association between hyperalgesia and the type of hoof injury has been reported (11). After injury, immune cells release pro-inflammatory cytokines at the site of injury, decreasing nociceptors threshold, leading to primary hyperalgesia (12–14). Several chronic painful conditions have reported elevated circulating levels of proinflammatory cytokines in humans (15). In lame cows diagnosed with interdigital dermatitis, Nazifi et al. (16) reported an increased in plasma TNF-α and IFN-γ. Additionally, pro-inflammatory cytokines can intensify the painful sensation acting directly in the CNS (17). Spinal cord cytokines can increase synaptic transmission toward supraspinal levels, enhancing pathological pain sensation and favoring central sensitization (18) which is a key element in chronic pain development and maintenance (19). Additionally, glial cells in the CNS react to signals associated with nociceptive transmission, morphologically changing into a reactive phenotype (20). Reactive microglia and astrocytes release cytokines, which in an autocrine and paracrine manner, facilitate neurons to glia and glia to glia communication, maintaining a state of neuroinflammation (21, 22). Moreover, the intrathecal administration of cytokines induces pain behavior in non-painful rodents (23); and the cerebrospinal concentration of cytokines correlates positively with pain intensity in humans with chronic pain (24). Although the role of proinflammatory cytokines in chronic pain is well-known, the spinal concentration of cytokines has not been reported in chronic lame cows. Here we hypothesize that chronic inflammatory lameness promotes persistent nociceptive input, which could induce changes in cytokine synthesis in the spinal cord. Therefore, the aim of this study was to determine the profile of cytokines in the spinal cord dorsal horn of dairy cows with painful chronic inflammatory lameness.

## Materials and Methods

The study and experimental protocol were reviewed and approved by the Ethics Committee for Animal Research of the Universidad Austral de Chile (N° 323/2018).

### Animals and Lameness Assessment

Animals were selected prospectively. Seven lame cows (Lame Group) were selected from a commercial dairy farm and seven non-lame cows (Non-Lame Group) were selected from a local slaughter-house. Selected animals included Holstein-Friesian (4) and Kiwi cross (10), weighing between 350 and 450 Kg with a parity range between 2 and 6. Inclusion criteria for lame cows included a history of chronic hind limb lameness caused by one of the most prevalent inflammatory hoof lesions previously described for Southern Chile, including white line disease, sole hemorrhage, heel erosion, sole ulcer and digital dermatitis (25). Animals were excluded if visible wounds, visible central or peripheral ataxia and acute or chronic mastitis were diagnosed. Lameness assessment was performed using a mobility scoring scale previously reported (26). Concisely, MS 0 not lame; MS 1 imperfect mobility/uneven; MS 2 impaired mobility/mildly lame; and MS 3 severely impaired/very lame. Only cows with the highest lameness score (MS = 3) were included in the lame group. Lame cows were euthanized at the dairy farm after general intravenous anesthesia and an intrathecal lidocaine injection at the atlanto-occipital foramen. Non-lame cows were euthanized at the slaughter-house by mechanically stunning and exsanguination following national regulations.

### Spinal Cord Sampling and Protein Extraction

Spinal cord samples were obtained immediately after euthanasia. Several lumbar spinal cord segments (L2–L4) were aseptically obtained after dissection of lumbar vertebrae. Bone tissue and meninges were removed, and spinal cord segments were thoroughly washed in cold phosphate-buffer saline (PBS), and then snap frozen in liquid nitrogen and stored at −80°C for further analysis. After unfreezing, dorsal horn segments were dissected, and 250 mg of tissue were homogenized in 1 mL of 4°C PBS using an Ultra Turrax tissue homogenizer at 4°C and 16,000 rpc three times for 30 s each. Homogenates were centrifuged at 20,000 x g for 10 min and supernatant was collected. Total protein concentration was quantified using the BSA Assay kit (Pierce^TM^ Thermo Scientific, Rochford USA).

### Multiplex Cytokine Assay

Cytokine analysis was performed using the Quantibody® Bovine Cytokine Array 1 kit (Ray Biotech) with an intra-assay coefficient of variation (CV) of <20%. The kit allows for simultaneous analysis of tumor necrosis factor-alpha (TNF-α), interleukin-1alpha (IL-1α), interleukin 13 (IL-13), chemokine-10 (CXCL10/IP-10), chemokine-9 (CXCL9/MIG), interferon-alpha (IFN-α), interferon-gamma (IFN-γ), interleukin-21 (IL-21), interleukin-36ra (IL-36ra), and macrophage inflammatory protein-1 beta (MIP-1β). A total of 150 μg of protein were loaded into each well with standards loaded similarly. Signal intensity was visualized using a laser scanner using a 532 nm filter). Experiments were performed in duplicate. Each standard curve of cytokine concentration was fitted using Graphpad Prism software.

### Statistical Analysis

Data are presented as mean ± SEM. Normality of the data was checked using the Shapiro-Wilk test. Differences between lame and non-lame cows for each cytokine were evaluated using the *t*-test. Overall alpha was set to *p* < 0.05. All analyses were performed using Graphpad Prism software (v7.0).

## Results

Spinal cord dorsal horn samples obtained from lame cows had higher concentrations of TNF-α (*p* = 0.024), IL-1-α (*p* = 0.0339), IL-13 (*p* = 0.0204), CXCL10 (*p* = 0.025), CXCL9 (*p* = 0.0252), IFN-α (*p* = 0.0391), and IFN-γ (*p* = 0.0027) compared to non-lame cows. IL-21 was the only cytokine with lower concentration in the spinal cord of lame cows in comparison to control cows (*p* = 0.0044). Spinal concentration of IL-36ra was not significantly increased (*p* = 0.2505) in lame cows, while MIP-1β concentration remained unchanged between groups (*p* = 0.9078). All results are summarized in Table 1.

**Table 1:** Spinal cord dorsal horn concentration of cytokines in Lame and Non-Lame cows.

	**Lame**	**Non-lame**	***P*-value**
TNF-α (pg/mL)	21.18 ± 5.41	5.02 ± 1.67	*P* = 0.0240
IL-1α (pg/mL)	4.78 ± 1.35	1.38 ± 0.45	*P* = 0.0339
IL-13 (pg/mL)	11.16 ± 1.35	5.06 ± 1.81	*P* = 0.0204
CXCL10 (pg/mL)	34.00 ± 5.61	16.54 ± 3.99	*P* = 0.0250
CXCL9 (pg/mL)	28.80 ± 6.69	9.04 ± 1.37	*P* = 0.0252
IFN-α (pg/mL)	69.14 ± 19.23	16.07 ± 4.39	*P* = 0.0391
IFN-γ (pg/mL)	3.10 ± 0.33	1.57 ± 0.09	*P* = 0.0027
IL-21 (pg/mL)	27.80 ± 4.47	55.78 ± 6.38	*P* = 0.0044
IL-36ra (pg/mL)	1.58 ± 0.53	0.90 ± 0.09	*P* = 0.2505
MIP-1β (pg/mL)	1.20 ± 0.14	1.23 ± 0.18	*P* = 0.9078

## Discussion

In this study we describe the profile of several cytokines in the dorsal horn of the spinal cord of dairy cows with chronic inflammatory lameness. Peripheral and Central Nervous (CNS) system cytokines contribute to central sensitization during chronic pain (18, 27, 28). Persistent pain leads to changes in the spinal cord including glial activation (21, 29). Neuropathic, inflammatory and neoplasic models of pain, and chronic opioid administration have shown to induce glial activation, increasing cytokine release, thus initiating and maintaining central sensitization (22). Additionally, resting microglia and astrocytes can be induced into a reactive state by pro-inflammatory cytokines. This cytokine-induced change in glia phenotype reveals that cytokines may have an autocrine/paracrine effect that facilitates neuron to glia or glia to glia communication during central sensitization (30). Moreover, cytokines are important modulators of neuronal functions (18, 31).

Spinal concentration of TNF-α was increased in lame cows. TNF-α is one of the most studied pro-inflammatory cytokines in the field of pain (32) and several experimental pain models have reported increased TNF-α expression in the CNS (22). It plays an important role in the development of central sensitization, mediating the expression and trafficking to the membrane of the AMPA receptor (31, 33), the phosphorylation of the NMDA receptor (34), and the increase in the frequency of spontaneous excitatory postsynaptic currents (EPSCs) (18). Zhang et al. (35) measured plasma TNF-α in transition cows before clinical signs of lameness, describing higher concentrations during the pre and postpartum, which suggested a state of clinical inflammation during that period. Similarly, Johnzon et al. (36) described an increased in TNF-α concentration both in plasma and milk after induction of mastitis in dairy cows. Although, the CSF concentration of TNF-α has not been reported in cows, our results are similar to those previously reported for human patients with chronic pain. Rozen and Swidan (37) described a marked increase in CSF and serum concentrations of TNF-α in human patients with persistent headache and chronic migraine. These results are in agreement with our finding in lame cows. Also, chronic inflammatory and neuropathic painful conditions have been associated with TNF-α secretion from reactive microglia (38, 39), which in turn promotes CXCL10 release from reactive astrocytes (40). TNF-α can also act as an autocrine factor in microglia, inducing the expression of several pro-inflammatory mediators (30, 40, 41).

IL-1α was increased in the spinal cord of lame cows. The role of IL-1α in inflammation has been extensively reported and recognized as an early trigger of the inflammatory response (42–45). Zhang et al. (35), described a state of subclinical inflammation during the prepartum of transition cows, which were characterized by lower concentrations of plasma IL-1α. Similar to our findings, higher concentrations of IL-1α have shown to occur in the spinal cord after dorsal root nerve compression (28) and in dorsal root ganglion (DRG) after peripheral nerve damage (46). Interestingly, both studies reported significant analgesia after the intrathecal administration of an IL-1α receptor antagonist. The potential role of IL-1α during chronic inflammation has been associated with the development of an inflammatory loop, triggered and maintained by IL-1α continuous secretion from damaged cells; which induces the autocrine and paracrine IL-1α expression from migrating cells with the subsequent expression of others cytokines and chemokines, including TNF-α, IL-8, IL-13, IL-6, CXCL9, and CXCL10 (42, 47, 48). We believe that the increased levels of cytokines detected in the spinal cord of lame cows could be the consequence of an inflammatory loop triggered by the augmented expression of IL-1α.

The spinal concentration of the anti-inflammatory cytokine IL-13 was increased in lame cows. IL-13 has been reported to increase in plasma, synovial fluid and muscular micro dialysate of patients with rheumatoid arthritis and jaw muscle pain, respectively (49, 50). The intraperitoneal administration of IL-13 induces marked analgesia in an inflammatory model of osteoarthritis and peritonitis (51), which confirms its anti-inflammatory properties. Similarly, the perineural injection of IL-13 reduced the pain threshold in rats with neuropathic pain (52). After injury and inflammation, neurons (53) and microglia (54) upregulate IL-13 in response to high levels of TNF-α and IFN-γ. This increase in IL-13 favors the downregulation of TNF-α, IL-1-β, and IFN-γ by macrophages (51, 55). Moreover, a neuroprotective role of IL-13 has been described (56). IL-13 induces apoptosis of reactive pro-inflammatory (M1) microglia (54, 57) or changes their phenotype into a protective type (M2) in order to suppress inflammation and promote healing (58).

Chemokines CXCL10 and CXCL9 were increased in the spinal cord of lame cows. Both chemokines are synthesized by immune cells in response to IFN-γ and TNF-α (59). IFN-γ-induced CXCL10 and CXCL9 expression has been reported in many inflammatory conditions of the CNS, including neuropathic pain (60–62). Accordingly, the *in vitro* administration of IFN-γ promotes microglial synthesis and release of CXCL10 and CXCL9 and astrocyte expression of CXCL-10 (63, 64). Therefore, the increased concentrations of spinal CXCL10 and CXCL9 in lame cows could indicate a response to high levels of IFN-γ and TNF-α. Nerve damage increases the spinal concentration of CXCL10 leading to hyperalgesia, which was successfully reverted after pharmacologic antagonism of its cognate receptor (CXC3R) (65). Also, the intrathecal injection of CXCL10 increased EPSCs and hyperalgesic behavior in rats (66). Recently, it has been proposed that CXCL10 plays an important role in microglia/astrocyte communication. In a bimodal inflammatory model in rats, a positive correlation between allodynia and microglial CXCL10/CXCR3 expression was followed by an increased astrocyte expression of CXCL10/CXCR3 (67).

In the present study, INF-α, a type I interferon member, was significantly upregulated in the spinal cord of lame cows. Its main role in the CNS is to be an important neuroprotective mechanism against viral infections (68). It is well-known that painful lameness in dairy cows is a consequence of peripheral inflammation (69). During peripheral inflammation, a marked increase in INF-α and its receptor in astrocytes and primary terminals afferents occurs (70). Interestingly, a significant interaction between INF-α and μ-opioids receptors has been documented, promoting analgesic properties of INF-α, which could explain some of its analgesic properties (71). Similarly, INF-α administered into the lateral ventricles in rats significantly reduced pain threshold (72) and its intrathecal administration attenuated hyperalgesic behavior in rats after hind paw injection of complete Freund's adjuvant (70). This analgesic INF-α dose was higher than the spinal concentration in lame and non-lame cows here reported, higher than the CSF concentration of human patients with CNS infection (73) and higher than those measured after traumatic brain injury (74). Prolonged INF-α therapy negatively impacts serotonin and monoamine function, stimulating excitatory transmission in rats (75, 76) and humans (77). According to Delgado (78), serotonergic and monoaminergic dysregulation have been proposed as a mechanism of chronic pain maintenance, reason why serotonin reuptake inhibitor drugs are frequently prescribed for treatment of chronic pain. Nonetheless, the INF-α effects over serotonergic transmission have not been studied thoroughly in chronic pain states. Although the increased spinal IFN-α concentration in lame cows here presented could be associated to an endogenous mechanism to control nociceptive transmission, a serotonergic dysregulation contributing to increased pain transmission cannot be completely ruled out.

IFN-γ is a type II pro-inflammatory cytokine that was increased in the spinal cord of lame cows. This result is similar to that described by Cuellar et al. (79), in which increased levels of IFN-γ were found in vertebral disc lavage samples from patients with chronic low back pain. It is known that neurons and astrocytes synthetize IFN-γ after nerve damage (80). Similarly, intrathecal administration of IFN-γ induces chronic pain behavior in rats (81) and systemic IFN-γ therapy in cancer patients induced spontaneous pain (82). Rats lacking the IFN-γ receptor did not express hyperalgesic behavior after nerve injury (81, 83).

IL-21 was the only downregulated cytokine in the spinal cord of lame cows. CD4^+^ T cells and natural killer T (NKT) cells produce IL-21, and augmented concentrations of brain IL-21 following experimental ischemia occurs after lymphocyte infiltration (84). A potential proinflammatory role of IL-21 in auto-immune and neurodegenerative diseases has been discussed (85). Interestingly, brain samples obtained from human patients with multiple sclerosis showed higher expression of IL-21 and its receptor, supporting a potential role of IL-21 in neurodegenerative diseases (86). Similarly, neuroinflammation promotes IL-21 expression by astrocytes (87). Although the role of IL-21 during chronic pain has not been thoroughly studied, Xue et al. (23) demonstrated that lumbar disc herniation induced an increased expression of IL-21, which positively correlated with pain intensity. Moreover, serum and synovial fluid from patients with rheumatoid arthritis showed high levels of IL-21 (88). In contrast, downregulation of IL-21 conferred resistance and neuroprotection against experimental brain ischemia in rats (84). According to this, it is possible that IL-21 downregulation in lame cows could reflect a protective response against neuroinflammation. However, more studies need to be performed in order to confirm this assumption.

Limitations of this study include the small sample size and a potential individual variability considering the multifactorial origin of bovine lameness. These aspects should be taken into account before results extrapolation. In this study, we were able to demonstrate that peripheral inflammation led to an increased concentration of cytokines in the dorsal horn of the spinal cord of chronically lame dairy cows. We believe that, as in humans, this state of neuroinflammation could have been initiated by sustained peripheral nociceptive transmission (89, 90). According to our results the impact of lameness in dairy cows is far beyond a local inflammation of the hoof, but also comprises inflammation of the CNS; with notorious and complex inflammatory events, involving IFN-γ, TNF-α, CXCL9, and CXCL10. The possible alteration in the spinal cytokine profile could indicate an orchestrated and self-perpetuated process in which IL-1α signaling pathway may play an important role. Additionally, the increased expression of anti-inflammatory cytokine IL-13, as well as the downregulation of IL-21 could indicate a physiological response in order to counteract the negative effects that prolonged inflammation of the spinal cord might exert on neurons and glia.

## Conclusion

We conclude that painful chronic inflammation of the hoof in dairy cows leads to a marked increase in cytokine concentration in the dorsal horn of the spinal cord, which could represent a state of neuroinflammation of the Central Nervous System.

## Data Availability Statement

The datasets generated for this study are available on request to the corresponding author.

## Ethics Statement

The animal study was reviewed and approved by Ethics Committee for Animal Research of the Universidad Austral de Chile. Written informed consent was obtained from the owners for the participation of their animals in this study.

## Author Contributions

DH and HB participated in the conceptualization, experimental design, and statistical analysis. DH, PS, and AR-R performed sample preparation, multiplex standardization, and analysis. DH prepared the manuscript. DH, MW, and HB critically reviewed the manuscript. All authors reviewed manuscript upon submission.

### Conflict of Interest

The authors declare that the research was conducted in the absence of any commercial or financial relationships that could be construed as a potential conflict of interest.

## References

[1] Cook NB Hess JP Foy MR Bennett TB Brotzman RL. Management characteristics, lameness, and body injuries of dairy cattle housed in high-performance dairy herds in Wisconsin. J Dairy Sci. (2016) 99:5879–91. 10.3168/jds.2016-10956 27132104

[2] Apkarian AV Scholz J Shared mechanisms between chronic pain and neurodegenerative disease. Drug Discov Today Dis Mech. (2006) 3:319–26. 10.1016/j.ddmec.2006.09.006

[3] Mogil JS. Animal models of pain: progress and challenges. Nat Rev Neurosci. (2009) 10:283–94. 10.1038/nrn2606 19259101

[4] Olsson IAS J Nicol C Niemi SM Sandøe P. From unpleasant to unbearable—why and how to implement an upper limit to pain and other forms of suffering in research with animals. ILAR J. (2020). 10.1093/ilar/ilz018. [Epub ahead of print]. 31996924

[5] Sadiq MB Ramanoon SZ Mossadeq WMS Mansor R Syed-Hussain SS Association between lameness and indicators of dairy cow welfare based on locomotion scoring, body and hock condition, leg hygiene and lying behavior. Animals. (2017) 7:79 10.3390/ani7110079 PMC5704108 29113033

[6] Warnick LD Janssen D Guard CL Gröhn YT. The effect of lameness on milk production in dairy cows. J Dairy Sci. (2001) 84:1988–97. 10.3168/jds.S0022-0302(01)74642-5 11573778

[7] Booth CJ Warnick LD Gröhn YT Maizon DO Guard CL Janssen D. Effect of lameness on culling in dairy cows. J Dairy Sci. (2004) 87:4115–22. 10.3168/jds.S0022-0302(04)73554-7 15545373

[8] Alawneh JI Laven RA Stevenson MA. The effect of lameness on the fertility of dairy cattle in a seasonally breeding pasture-based system. J Dairy Sci. (2011) 94:5487–93. 10.3168/jds.2011-4395 22032371

[9] Whay HR Waterman AE Webster AJF O'Brien JK. The influence of lesion type on the duration of hyperalgesia associated with hindlimb lameness in dairy cattle. Vet J. (1998) 156:23–9. 10.1016/S1090-0233(98)80058-0 9691848

[10] Dubuisson D Dennis SG. The formalin test: a quantitative study of the analgesic effects of morphine, meperidine, and brain stem stimulation in rats and cats. Pain. (1977) 4:161–74. 10.1016/0304-3959(77)90130-0 564014

[11] Tadich N Tejeda C Bastias S Rosenfeld C Green LE. Nociceptive threshold, blood constituents and physiological values in 213 cows with locomotion scores ranging from normal to severely lame. Vet J. (2013) 197:401–5. 10.1016/j.tvjl.2013.01.029 23499542

[12] Sommer C Kress M. Recent findings on how proinflammatory cytokines cause pain: peripheral mechanisms in inflammatory and neuropathic hyperalgesia. Neurosci Lett. (2004) 361:184–7. 10.1016/j.neulet.2003.12.007 15135924

[13] Woolf CJ Ma Q. Nociceptors-noxious stimulus detectors. Neuron. (2007) 55:353–64. 10.1016/j.neuron.2007.07.016 17678850

[14] Kress M Nociceptor sensitization by proinflammatory cytokines and chemokines. Open Pain J. (2014) 3:97–107. 10.2174/1876386301003010097

[15] Klyne DM Barbe MF Hodges PW. Systemic inflammatory profiles and their relationships with demographic, behavioural and clinical features in acute low back pain. Brain Behav Immun. (2017) 60:84–92. 10.1016/j.bbi.2016.10.003 27720935

[16] Nazifi S Esmailnezhad Z Haghkhah M Ghadirian S Mirzaei A. Acute phase response in lame cattle with interdigital dermatitis. World J Microbiol Biotechnol. (2012) 28:1791–6. 10.1007/s11274-011-0995-9 22805961

[17] DeLeo JA Yezierski RP. The role of neuroinflammation and neuroimmune activation in persistent pain. Pain. (2001) 90:1–6. 10.1016/S0304-3959(00)00490-5 11166964

[18] Kawasaki Y Zhang L Cheng JK Ji RR. Cytokine mechanisms of central sensitization: distinct and overlapping role of interleukin-1beta, interleukin-6, and tumor necrosis factor-alpha in regulating synaptic and neuronal activity in the superficial spinal cord. J Neurosci. (2008) 28:5189–94. 10.1523/JNEUROSCI.3338-07.2008 18480275 PMC2408767

[19] Latremoliere A Woolf CJ. Central sensitization: a generator of pain hypersensitivity by central neural plasticity. J Pain. (2009) 10:895–926. 10.1016/j.jpain.2009.06.012 19712899 PMC2750819

[20] Raghavendra V Tanga FY DeLeo JA Complete freunds adjuvant-induced peripheral inflammation evokes glial activation and proinflammatory cytokine expression in the CNS. *Eur J Neurosci*. (2004) 20:467–73. 10.1111/j.1460-9568.2004.03514.x 15233755

[21] Watkins LR Milligan ED Maier SF. Glial activation: a driving force for pathological pain. Trends Neurosci. (2001) 24:450–5. 10.1016/S0166-2236(00)01854-3 11476884

[22] Ji RR Berta T Nedergaard M. Glia and pain: is chronic pain a gliopathy? Pain. (2013) 154:S10–28. 10.1016/j.pain.2013.06.022 23792284 PMC3858488

[23] Xue H Yao Y Wang X Zhang F Jiang X Liu J. Interleukin-21 is associated with the pathogenesis of lumbar disc herniation. Iran J Allergy Asthma Immunol. (2015) 14:509–18. 26742440

[24] Sainoh T Inage K Orita S Koda M Furuya T Yamauchi K. Correlation among inflammatory cytokine expression levels, degree of disk degeneration, and predominant clinical symptoms in patients with degenerated intervertebral discs. Asian Spine J. (2017) 11:472–7. 10.4184/asj.2017.11.3.472 28670416 PMC5481603

[25] Tadich N Flor E Green L. Associations between hoof lesions and locomotion score in 1098 unsound dairy cows. Vet J. (2010) 184:60–5. 10.1016/j.tvjl.2009.01.005 19211281

[26] Whay HR Main DCJ Green LE Webster AJF. Assessment of the welfare of dairy cattle using animal-based measurements: direct observations and investigation of farm records. Vet Rec. (2003) 153:197–202. 10.1136/vr.153.7.197 12956296

[27] Clark AK Old EA Malcangio M. Neuropathic pain and cytokines: current perspectives. J Pain Res. (2013) 6:803–14. 10.2147/JPR.S53660 24294006 PMC3839806

[28] Rothman SM Winkelstein BA. Cytokine antagonism reduces pain and modulates spinal astrocytic reactivity after cervical nerve root compression. Ann Biomed Eng. (2010) 38:2563–76. 10.1007/s10439-010-0012-8 20309734

[29] Milligan ED Watkins LR. Pathological and protective roles of glia in chronic pain. Nat Rev Neurosci. (2009) 10:23–36. 10.1038/nrn2533 19096368 PMC2752436

[30] Hanisch UK. Microglia as a source and target of cytokines. Glia. (2002) 40:140–55. 10.1002/glia.10161 12379902

[31] Beattie EC Stellwagen D Morishita W Bresnahan JC Byeong KH Von Zastrow M. Control of synaptic strength by glial TNFalpha. Science. (2002) 295:2282–5. 10.1126/science.1067859 11910117

[32] Leung L Cahill CM. TNF-alpha and neuropathic pain – a review. J Neuroinflammation. (2010) 7:27. 10.1186/1742-2094-7-27 20398373 PMC2861665

[33] Wigerblad G Huie JR Yin HZ Leinders M Pritchard RA Koehrn FJ. Inflammation-induced GluA1 trafficking and membrane insertion of Ca^2+^ permeable AMPA receptors in dorsal horn neurons is dependent on spinal tumor necrosis factor, PI3 kinase and protein kinase A. Exp Neurol. (2017) 293:144–58. 10.1016/j.expneurol.2017.04.004 28412220 PMC5539914

[34] Wei F Guo W Zou S Ren K Dubner R. Supraspinal glial-neuronal interactions contribute to descending pain facilitation. J Neurosci. (2008) 28:10482–95. 10.1523/JNEUROSCI.3593-08.2008 18923025 PMC2660868

[35] Zhang G Hailemariam D Dervishi E Deng Q Goldansaz SA Dunn SM. Alterations of innate immunity reactants in transition dairy cows before clinical signs of lameness. Animals. (2015) 5:717–47. 10.3390/ani5030381 26479383 PMC4598703

[36] Johnzon CF Dahlberg J Gustafson AM Waern I Moazzami AA Östensson K. The effect of lipopolysaccharide-induced experimental bovine mastitis on clinical parameters, inflammatory markers, and the metabolome: a kinetic approach. Front Immunol. (2018) 9:1487. 10.3389/fimmu.2018.01487 29988549 PMC6026673

[37] Rozen T Swidan SZ. Elevation of CSF tumor necrosis factor α levels in new daily persistent headache and treatment refractory chronic migraine. Headache. (2007) 47:1050–5. 10.1111/j.1526-4610.2006.00722.x 17635596

[38] Xu JT Xin WJ Zang Y Wu CY Liu XG. The role of tumor necrosis factor-alpha in the neuropathic pain induced by lumbar 5 ventral root transection in rat. Pain. (2006) 123:306–21. 10.1016/j.pain.2006.03.011 16675114

[39] Zhang L Berta T Xu ZZ Liu T Park JY Ji RR. TNF-alpha contributes to spinal cord synaptic plasticity and inflammatory pain: distinct role of TNF receptor subtypes 1 and 2. Pain. (2011) 152:419–27. 10.1016/j.pain.2010.11.014 21159431 PMC3022092

[40] Choi SS Lee HJ Lim I Satoh JI Kim SU. Human astrocytes: secretome profiles of cytokines and chemokines. PLoS ONE. (2014) 9:e92325. 10.1371/journal.pone.0092325 24691121 PMC3972155

[41] Kuno R Wang J Kawanokuchi J Takeuchi H Mizuno T Suzumura A. Autocrine activation of microglia by tumor necrosis factor-α. J Neuroimmunol. (2005) 162:89–96. 10.1016/j.jneuroim.2005.01.015 15833363

[42] Di Paolo NC Shayakhmetov DM. Interleukin 1α and the inflammatory process. Nat Immunol. (2016) 17:906–13. 10.1038/ni.3503 27434011 PMC5152572

[43] Turner NA Das A Warburton P O'Regan DJ Ball SG Porter KE. Interleukin-1alpha stimulates proinflammatory cytokine expression in human cardiac myofibroblasts. Am J Physiol Hear Circ Physiol. (2009) 297:H1117–27. 10.1152/ajpheart.00372.2009 19648252

[44] Luheshi NM Kovács KJ Lopez-Castejon G Brough D Denes A. Interleukin-1α expression precedes IL-1β after ischemic brain injury and is localised to areas of focal neuronal loss and penumbral tissues. J Neuroinflammation. (2011) 8:186. 10.1186/1742-2094-8-186 22206506 PMC3259068

[45] Rider P Carmi Y Guttman O Braiman A Cohen I Voronov E. IL-1α and IL-1β Recruit different myeloid cells and promote different stages of sterile inflammation. J Immunol. (2011) 187:4835–43. 10.4049/jimmunol.1102048 21930960

[46] Mika J Korostynski M Kaminska D Wawrzczak-Bargiela A Osikowicz M Makuch W Interleukin-1alpha has antiallodynic and antihyperalgesic activities in a rat neuropathic pain model. Pain. (2008) 138:587–97. 10.1016/j.pain.2008.02.015 18374486

[47] Dube PH Revell PA Chaplin DD Lorenz RG Miller VL A role for IL-1α in inducing pathologic inflammation during bacterial infection. Proc Natl Acad Sci USA. (2001) 98:10880–85. 10.1073/pnas.191214498 11526216 PMC58568

[48] Pugazhenthi S Zhang Y Bouchard R Mahaffey G. Induction of an inflammatory loop by interleukin-1β and tumor necrosis factor-α involves NF-kB and STAT-1 in differentiated human neuroprogenitor cells. PLoS ONE. (2013) 8:e69585. 10.1371/journal.pone.0069585 23922745 PMC3726669

[49] Imamura M Targino RA Hsing WT Imamura S Azevedo RS Villas Boas LS. Concentration of cytokines in patients with osteoarthritis of the knee and fibromyalgia. Clin Interv Aging. (2014) 9:939–44. 10.2147/CIA.S60330 24959074 PMC4061171

[50] Louca Jounger S Christidis N Svensson P List T Ernberg M. Increased levels of intramuscular cytokines in patients with jaw muscle pain. J Headache Pain. (2017) 18:30. 10.1186/s10194-017-0737-y 28243900 PMC5328896

[51] Vale ML Marques JB Moreira CA Rocha FAC Ferreira SH Poole S. Antinociceptive effects of interleukin-4,−10, and−13 on the writhing response in mice and zymosan-induced knee joint incapacitation in rats. J Pharmacol Exp Ther. (2003) 304:102–8. 10.1124/jpet.102.038703 12490580

[52] Kiguchi N Sakaguchi H Kadowaki Y Saika F Fukazawa Y Matsuzaki S. Peripheral administration of interleukin-13 reverses inflammatory macrophage and tactile allodynia in mice with partial sciatic nerve ligation. J Pharmacol Sci. (2017) 133:53–6. 10.1016/j.jphs.2016.11.005 28057412

[53] Yu JT Lee CH Yoo KY Choi JH Li H Park OK Maintenance of anti-inflammatory cytokines and reduction of glial activation in the ischemic hippocampal CA1 region preconditioned with lipopolysaccharide. J Neurol Sci. (2010) 49:2214–28. 10.1016/j.jns.2010.06.004 20580380

[54] Shin WH Lee DY Park KW Kim SU Yang MS Joe EH. Microglia expressing interleukin-13 undergo cell death and contribute to neuronal survival *in vivo*. Glia. (2004) 46:142–52. 10.1002/glia.10357 15042582

[55] Albanesi C Fairchild HR Madonna S Scarponi C De Pità O Leung DYM. IL-4 and IL-13 negatively regulate TNF-alpha- and IFN-gamma-induced beta-defensin expression through STAT-6, suppressor of cytokine signaling (SOCS)-1, and SOCS-3. J Immunol. (2007) 179:984–92. 10.4049/jimmunol.179.2.984 17617590

[56] Mori S Maher P Conti B. Neuroimmunology of the interleukins 13 and 4. Brain Sci. (2016) 6:18. 10.3390/brainsci6020018 27304970 PMC4931495

[57] Yang MS Park EJ Sohn S Kwon HJ Shin WH Pyo HK. Interleukin-13 and -4 induce death of activated microglia. Glia. (2002) 38:273–80. 10.1002/glia.10057 12007140

[58] Guglielmetti C Le Blon D Santermans E Salas-Perdomo A Daans J De Vocht N. Interleukin-13 immune gene therapy prevents CNS inflammation and demyelination via alternative activation of microglia and macrophages. Glia. (2016) 64:2181–200. 10.1002/glia.23053 27685637

[59] Benveniste EN Benos DJ. TNF-alpha- and IFN-gamma-mediated signal transduction pathways: effects on glial cell gene expression and function. FASEB J. (1995) 9:1577–84. 10.1096/fasebj.9.15.8529837 8529837

[60] Müller M Carter S Hofer MJ Campbell IL. Review: the chemokine receptor CXCR3 and its ligands CXCL9, CXCL10 and CXCL11 in neuroimmunity - a tale of conflict and conundrum. Neuropathol Appl Neurobiol. (2010) 36:368–87. 10.1111/j.1365-2990.2010.01089.x 20487305

[61] Roberts WK Blachère NE Frank MO Dousmanis A Ransohoff RM Darnell RB. A destructive feedback loop mediated by CXCL10 in central nervous system inflammatory disease. Ann Neurol. (2015) 78:619–29. 10.1002/ana.24494 26224283 PMC4583819

[62] Bäckryd E Lind AL Thulin M Larsson A Gerdle B Gordh T. High levels of cerebrospinal fluid chemokines point to the presence of neuroinflammation in peripheral neuropathic pain: a cross-sectional study of 2 cohorts of patients compared with healthy controls. Pain. (2017) 158:2487–95. 10.1097/j.pain.0000000000001061 28930774 PMC5690569

[63] Carter SL Müller M Manders PM Campbell IL. Induction of the genes for Cxcl9 and Cxcl10 is dependent on IFN-gamma but shows differential cellular expression in experimental autoimmune encephalomyelitis and by astrocytes and microglia *in vitro*. Glia. (2007) 55:1728–39. 10.1002/glia.20587 17902170

[64] Ellis SL Gysbers V Manders PM Li W Hofer MJ Müller M. The cell-specific induction of CXC chemokine ligand 9 mediated by IFN-gamma in microglia of the central nervous system is determined by the myeloid transcription factor PU.1. J Immunol. (2010) 185:1864–77. 10.4049/jimmunol.1000900 20585034 PMC2925661

[65] Chen Y Yin D Fan B Zhu X Chen Q Li Y. Chemokine CXCL10/CXCR3 signaling contributes to neuropathic pain in spinal cord and dorsal root ganglia after chronic constriction injury in rats. Neurosci Lett. (2019) 694:20–8. 10.1016/j.neulet.2018.11.021 30448292

[66] Wu XB He LN Jiang BC Shi H Bai XQ Zhang WW Spinal CXCL9 and CXCL11 are not involved in neuropathic pain despite an upregulation in the spinal cord following spinal nerve injury. Mol Pain. (2018) 14:1744806918777401 10.1177/1744806918777401 29712506 PMC5967156

[67] Yu Q Tian DL Tian Y Zhao XT Yang XY. Elevation of the chemokine pair CXCL10/CXCR3 initiates sequential glial activation and crosstalk during the development of bimodal inflammatory pain after spinal cord ischemia reperfusion. Cell Physiol Biochem. (2018) 49:2214–28. 10.1159/000493825 30257241

[68] Hwang M Bergmann CC. Alpha/Beta interferon (IFN-α/β) signaling in astrocytes mediates protection against viral encephalomyelitis and regulates IFN-γ-dependent responses. J Virol. (2018) 92:e01901–17. 10.1128/JVI.01901-17 29491163 PMC5923078

[69] Rodríguez AR Olivares FJ Descouvieres PT Werner MP Tadich NA Bustamante HA Thermographic assessment of hoof temperature in dairy cows with different mobility scores. Livest Sci. (2016) 184:92–6. 10.1016/j.livsci.2015.12.006

[70] Liu CC Gao YJ Luo H Berta T Xu ZZ Ji RR. Interferon alpha inhibits spinal cord synaptic and nociceptive transmission via neuronal-glial interactions. Sci Rep. (2016) 6:34356. 10.1038/srep34356 27670299 PMC5037469

[71] Dafny N Yang PB. Interferon and the central nervous system. Eur J Pharmacol. (2005) 523:1–15. 10.1016/j.ejphar.2005.08.029 16226745

[72] Jiang CL Song LX Lu CL You ZD Wang YX Sun LY. Analgesic effect of interferon-alpha via mu opioid receptor in the rat. Neurochem Int. (2000) 36:193–6. 10.1016/S0197-0186(99)00124-2 10676852

[73] Rodero MP Decalf J Bondet V Hunt D Rice GI Werneke S. Detection of interferon alpha protein reveals differential levels and cellular sources in disease. J Exp Med. (2017) 214:1547–55. 10.1084/jem.20161451 28420733 PMC5413335

[74] Helmy A Carpenter KLH Menon DK Pickard JD Hutchinson PJA. The cytokine response to human traumatic brain injury: temporal profiles and evidence for cerebral parenchymal production. J Cereb Blood Flow Metab. (2011) 31:658–70. 10.1038/jcbfm.2010.142 20717122 PMC3049520

[75] Zahiu CDM Rimbas M. Neuropsychiatric side-effects of interferon-alpha treatment: pathophysiology and therapeutic options. Maedica. (2014) 9:121–6. 25705266 PMC4296753

[76] Paul S Ricour C Sommereyns C Sorgeloos F Michiels T. Type I interferon response in the central nervous system. Biochimie. (2007) 89:770–8. 10.1016/j.biochi.2007.02.009 17408841

[77] Haroon E Woolwine BJ Chen X Pace TW Parekh S Spivey JR. IFN-alpha-induced cortical and subcortical glutamate changes assessed by magnetic resonance spectroscopy. Neuropsychopharmacology. (2014) 39:1777–85. 10.1038/npp.2014.25 24481242 PMC4023151

[78] Delgado PL. Serotonin noradrenaline reuptake inhibitors: new hope for the treatment of chronic pain. Int J Psychiatry Clin Pract. (2006) 10(Suppl. 2):16–21. 10.1080/13651500600637098 24921678

[79] Cuellar JM Golish SR Reuter MW Cuellar VG Angst MS Carragee EJ. Cytokine evaluation in individuals with low back pain using discographic lavage. Spine J. (2010) 10:212–8. 10.1016/j.spinee.2009.12.007 20207331

[80] Racz I Nadal X Alferink J Baños JE Rehnelt J Martín M Interferon-gamma is a critical modulator of CB2 cannabinoid receptor signaling during neuropathic pain. J Neurosci. (2008) 28:12136–45. 10.1523/JNEUROSCI.3402-08.2008 19005078 PMC3844840

[81] Tsuda M Masuda T Kitano J Shimoyama H Tozaki-Saitoh H Inoue K. IFN-gamma receptor signaling mediates spinal microglia activation driving neuropathic pain. Proc Natl Acad Sci USA. (2009) 106:8032–7. 10.1073/pnas.0810420106 19380717 PMC2683100

[82] Mahmoud HH Pui CH Kennedy W Jaffe HS Crist WM Murphy SB. Phase I study of recombinant human interferon gamma in children with relapsed acute leukemia. Leukemia. (1992) 6:1181–4. 1434801

[83] Moritz KE McCormack NM Abera MB Viollet C Yauger YJ Sukumar G. The role of the immunoproteasome in interferon-γ-mediated microglial activation. Sci Rep. (2017) 7:9365. 10.1038/s41598-017-09715-y 28839214 PMC5571106

[84] Clarkson BDS Ling C Shi Y Harris MG Rayasam A Sun D. T cell-derived interleukin (IL)-21 promotes brain injury following stroke in mice. J Exp Med. (2014) 211:595–604. 10.1084/jem.20131377 24616379 PMC3978271

[85] Ghalamfarsa G Mahmoudi M Mohammadnia-Afrouzi M Yazdani Y Anvari E Hadinia A. IL-21 and IL-21 receptor in the immunopathogenesis of multiple sclerosis. J Immunotoxicol. (2016) 13:274–85. 10.3109/1547691X.2015.1089343 26507681

[86] Tzartos JS Craner MJ Friese MA Jakobsen KB Newcombe J Esiri MM. IL-21 and IL-21 receptor expression in lymphocytes and neurons in multiple sclerosis brain. Am J Pathol. (2011) 178:794–802. 10.1016/j.ajpath.2010.10.043 21281812 PMC3032888

[87] Xiong XY Wang TG Yang MH Meng ZY Yang QW Wang FX. Interleukin-21 expression in hippocampal astrocytes is enhanced following kainic acid-induced seizures. Neurol Res. (2016) 38:151–7. 10.1080/01616412.2015.1135557 27118610

[88] Dinesh P Rasool M. Multifaceted role of IL-21 in rheumatoid arthritis: current understanding and future perspectives. J Cell Physiol. (2018) 233:3918–28. 10.1002/jcp.26158 28833093

[89] Chen WW Zhang X Huang WJ. Role of neuroinflammation in neurodegenerative diseases (Review). Mol Med Rep. (2016) 13:3391–6. 10.3892/mmr.2016.4948 26935478 PMC4805095

[90] Lurie DI. An integrative approach to neuroinflammation in psychiatric disorders and neuropathic pain. J Exp Neurosci. (2018) 12:1179069518793639. 10.1177/1179069518793639 30127639 PMC6090491

